# Therapeutic Potential of d-MAPPS™ for Ocular Inflammatory Diseases and Regeneration of Injured Corneal and Retinal Tissue

**DOI:** 10.3390/ijms232113528

**Published:** 2022-11-04

**Authors:** Carl Randall Harrell

**Affiliations:** Regenerative Processing Plant, LLC, 34176 US Highway 19 N Palm Harbor, Palm Harbor, FL 34684, USA; dr.carlharrell@gmail.com or dr.harrell@regenerativeplant.org

**Keywords:** d-MAPPS™, eye inflammation, corneal injury, retinal injury

## Abstract

The invasion of microbial pathogens and/or sterile inflammation caused by physical/chemical injuries, increased ocular pressure, oxidative stress, and ischemia could lead to the generation of detrimental immune responses in the eyes, which result in excessive tissue injury and vision loss. The bioavailability of eye drops that are enriched with immunoregulatory and trophic factors which may concurrently suppress intraocular inflammation and promote tissue repair and regeneration is generally low. We recently developed “derived- Multiple Allogeneic Proteins Paracrine Signaling regenerative biologics platform technology d-MAPPS™”, a bioengineered biological product which is enriched with immunomodulatory and trophic factors that can efficiently suppress detrimental immune responses in the eye and promote the repair and regeneration of injured corneal and retinal tissues. The results obtained in preclinical and clinical studies showed that d-MAPPS™ increased the viability of injured corneal cells, inhibited the production of inflammatory cytokines in immune cells, alleviated inflammation, and restored vision loss in patients suffering from meibomian gland dysfunction and dry eye disease. Herewith, we emphasized molecular mechanisms responsible for the therapeutic efficacy of d-MAPPS™ and we presented the main beneficial effects of d-MAPPS™ in clinical settings, indicating that the topical administration of d-MAPPS™ could be considered a new therapeutic approach for the treatment of ocular inflammatory diseases and for the repair and regeneration of injured corneal and retinal tissues.

## 1. Introduction

The eye is considered to be an immune-privileged site [1]. Under physiological conditions, vasculature of the eye is limited to regions which are positioned outside of the central light path [1]. The invasion of microbial pathogens and/or sterile inflammation caused by physical/chemical injuries, increased ocular pressure, oxidative stress, and ischemia result in the generation of an immune response which is elicited in order to prevent excessive tissue injury and vision loss [2]. Response to microbial-pathogen-associated molecular patterns (PAMPs) and the massive release of alarmins and damage-associated molecular patterns (DAMPs) from injured cells lead to the activation of innate immune cells in the eye [2].

Professional antigen-presenting cells (macrophages and dendritic cells (DCs)) recognize PAMPs and DAMPs, capture and phagocyte microbial pathogens and necrotic cells, produce inflammatory chemokines and cytokines, and elicit inflammation in the eyes [3,4]. Upon the phagocytosis of foreign pathogens, macrophages obtain pro-inflammatory M1 phenotype and produce large amounts of inflammatory chemokines (chemokine (C-C motif) ligand (CCL)6, CCL2, and CCL20) and cytokines (tumor necrosis factor alpha (TNF-α) and interleukin (IL)-1β) which induce the increased expression of E and P selectins on endothelial cells (ECs), enabling the massive influx of immune cells in injured eyes [3]. Activated DCs crucially contribute to the generation of an adaptive immune response in inflamed eyes because they present peptide antigens from phagocyted cells and microbial pathogens to the naïve CD4+ and CD8+ T cells in the regional lymph nodes [4]. Antigen-primed DCs, through the production of pro-Th1 (IL-12, interferon gamma (IFN-γ)) and pro-Th17 cytokines (IL-1β, IL-6, IL-23, and transforming growth factor beta (TGF-β)), induce the generation and proliferation of inflammatory Th1 and Th17 cells [4].

Th17-cell-sourced IL-17 activates pro-inflammatory N1 neutrophils in the eyes, whereas Th1-cell-derived IFN-γ induces the activation of pro-inflammatory M1 macrophages [5]. N1 neutrophils and M1 macrophages release tissue-damaging nitric oxide (NO) and reactive oxygen species (ROS) and produce inflammatory chemokines and cytokines that attract circulating leukocytes in injured and inflamed eyes, enabling generation of the “inflammatory loop” which results in the aggravation of ongoing inflammation [4,5]. The cross-talk between activated DCs, M1 macrophages, N1 neutrophils, and Th1 and Th17 lymphocytes is crucial for the development of a detrimental immune response in inflamed eyes [4].

Eye injury and consequent inflammation may occur in the anterior and posterior segments of the eyes and could result in the development of various pathologies with different clinical manifestations [6,7]. Grittiness, sensitivity to light, watery eyes, scratchiness, redness, foreign body sensation, pain, and blurred vision are the most common symptoms that occur as a consequence of on-going inflammatory response in the eye and are observed in patients suffering from anterior uveitis, scleritis and keratitis [6,7]. Dryness, burning or scratchy sensations in eyes, stringy mucus in or around the eyes, foreign body sensations, and difficulty wearing contact lenses are frequently reported by patients with dry eye disease (DED), and are consequences of increased tear film osmolarity due to the inflammation-induced meibomian gland dysfunction (MGD) [4]. Significantly reduced functional visual acuity impairs the performance of vision-dependent daily activities (such as reading, writing, and driving) and greatly diminishes the quality of life of patients suffering from inflammatory eye diseases [4]. Loss of night and peripheral vision are usually reported by patients suffering from retinitis pigmentosa (RP) and may progress to complete vision loss [8]. Blurred vision and blindness may be a consequence of aggravated and untreated inflammatory and degenerative eye diseases; therefore, patients suffering from these disorders should receive time-appropriate and disease-specific treatment which will efficiently attenuate ongoing inflammation and promote the repair and regeneration of injured cells in affected eyes [4]. Current therapeutic approaches used in the treatment of the majority of eye disorders are directed towards improving the symptoms rather than towards eliminating the cause of disease [9]. The topical administration of immunosuppressive eye drops is usually recommended for the attenuation of inflammatory and autoimmune eye diseases, whereas the administration of neurotrophins in deep structures of the eyes has beneficial effects in the therapy of degenerative eye diseases [9]. However, well-developed protective mechanisms of the eye ensure the rapid clearance of eye drops from the pre-corneal space, limiting ocular penetration of the incorporated drug [9,10]. Moreover, the bioavailability of eye drops that are enriched with immunoregulatory and trophic factors which may suppress inflammation and promote tissue regeneration is generally low [10]. Additionally, the long-term, systemic use of immunosuppressive drugs may result in the development of severe, secondary immunodeficiency, significantly increasing the risk for the development of infectious diseases and malignancy [9,10]. Therefore, there is an urgent need for the development and clinical use of newly generated eye drops which will contain growth factors and immunomodulatory molecules that will be able to bypass ocular surface barrier and to reach the target parenchymal and immune cells in the eyes of patients suffering from inflammatory and degenerative eye diseases [9].

Human amniotic fluid (AF) contains many immunosuppressive factors and is able to efficiently attenuate ongoing inflammation [11]. It plays an important role in the defense of fetal and uterine structures against infection and can modulate the maternal immune response against fetal antigens, preventing rejection of the fetus [11]. Purified AF is a non-antigeneic solution which does not elicit a detrimental immune response; thus, it represents a valuable source for the development of immunomodulatory drugs [11]. In line with these observations, we recently developed “derived- Multiple Allogeneic Proteins Paracrine Signaling d-MAPPS™ regenerative biologics platform technology”, which is a bioengineered biological product derived from human amniotic fluid (AF), manufactured under current Good Manufacturing Practices (cGMP), regulated and reviewed by the Food and Drug Administration (FDA) [11]. The therapeutic potential of d-MAPPS™ in the treatment of inflammatory eye diseases and in corneal regeneration has been documented in several in vitro studies and pilot clinical trials, indicating that it could be considered a new therapeutic agent in regenerative ophthalmology [11,12,13,14,15,16]. Herewith, we emphasized the molecular mechanisms responsible for the therapeutic efficacy of d-MAPPS™ and present the main beneficial effects of d-MAPPS™ which have been observed in clinical settings.

## 2. Results

### 2.1. Therapeutic Potential of d-MAPPS™ in the Repair and Regeneration of Injured Corneal Epithelium

**Figure 1:**
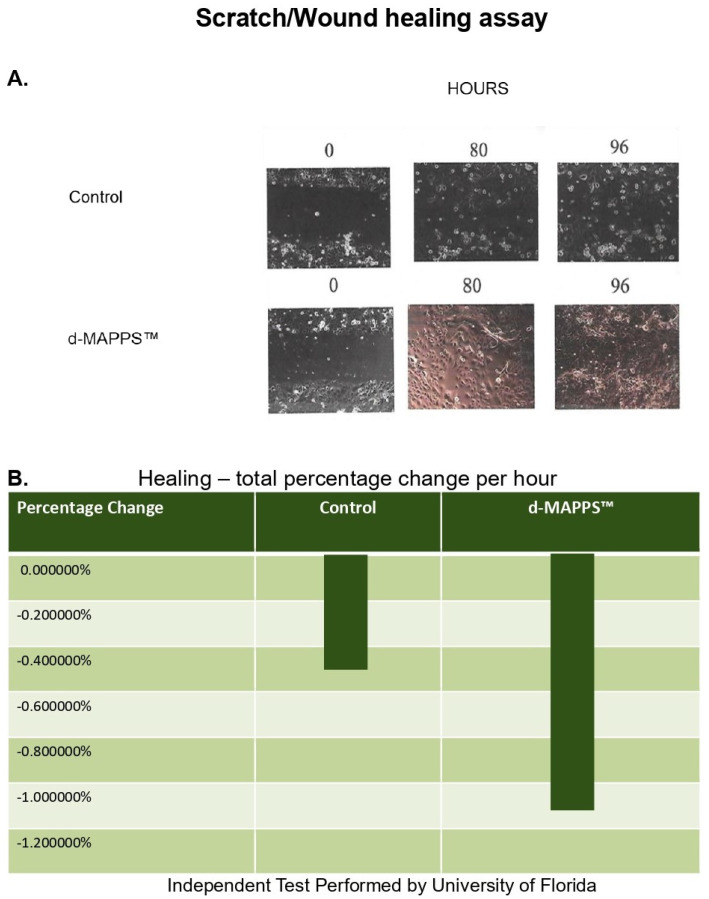
Therapeutic potential of d-MAPPS™ in the treatment of corneal wounds. (**A**) Cell-to-cell contact of the human corneal epithelial cell was completely restored after 96 h of d-MAPPS™-based treatment. (**B**) Scratch/wound healing assay showed that d-MAPPS™ significantly improved the proliferative and migratory properties of human corneal epithelial cells in vitro, indicating the therapeutic potential of d-MAPPS™ in the treatment of corneal wounds.

d-MAPPS™ improved survival and restored the morphology of benzalkonium chloride (BAK)-treated human corneal epithelial cells (HCECs) [13]. BAK is a quaternary ammonium compound which is widely used as a preservative in eye drops [17]. BAK alters the morphology of HCEC, modulates signaling pathways which regulate the HCEC cycle, affects the release of adenosine three phosphate (ATP), induces oxidative stress in HCECs, and induces direct cytotoxic effects by promoting the activation of caspase-3-dependent apoptosis in injured HCECs [17]. BAK-treated HCECs which grew under the presence of d-MAPPS™ restored their morphology and function [13]. Loss of cell-to-cell contact was not observed in BAK+ d-MAPPS™-treated HCECs which grew in the same manner as under standard culture conditions. Importantly, d-MAPPS™ significantly improved the viability of BAK-injured HCEC and restored their protective and immunoregulatory functions [13]. As evidenced by scratch/wound healing assay (the most commonly used method for studying cell migration and proliferation patterns in vitro) [13], researchers from the University of Florida revealed that d-MAPPS™ significantly improved the migratory and proliferative properties of HCEC, resulting in the complete restoration of corneal wounds induced in vitro. Cell-to-cell contacts of HCECs were completely restored after 96 h of d-MAPPS™-based treatment [13], indicating the therapeutic potential of d-MAPPS™ in the treatment of corneal wounds (Figure 1).

**Figure 2:**
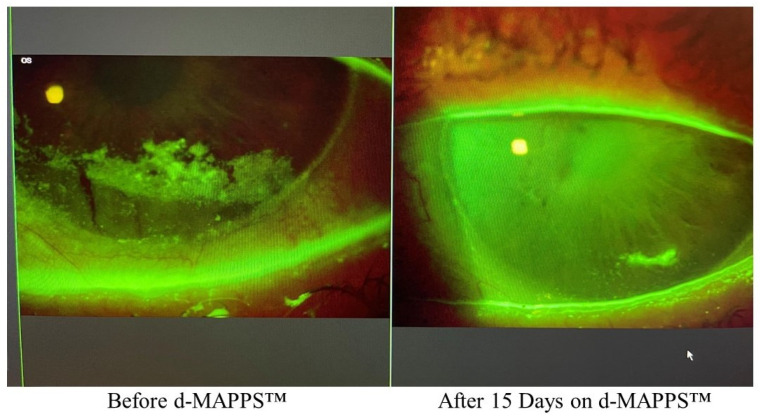
Therapeutic potential of d-MAPPS™ in the treatment of neurotrophic keratitis. Significant improvements in neurotrophic keratitis were observed in an 80-year-old patient who received d-MAPPS™ for 15 days (3–4 times per day for 4 days, then 2–3 times a day).

In addition to its direct cytotoxic effects, BAK induces the enhanced expression of *CCL2*, *IL-6*, and *MIF* genes, resulting in the increased recruitment of circulating monocytes and lymphocytes in injured eyes [18]. The BAK-dependent injury of HCEC is followed by the massive release of alarmins from damaged HCECs, which results in the activation of DAMP receptors on recruited immune cells [18]. Therefore, corneal injuries usually result in the development of corneal inflammation: keratitis [18]. IL-1β plays the most important pathogenic role in the progression of keratitis [19]. During the early stage of corneal damage, injured HCECs secrete high amounts of IL-1β, which is stored in HCECs and released when the cell membrane is damaged by external insults. Subsequently, IL-1β binds to its receptor (IL-1R) on EC, initiating an inflammatory cascade which finally results in the massive influx of circulating monocytes and lymphocytes in injured and inflamed corneas [19].

d-MAPPS™ contains a high concentration of interleukin 1 receptor antagonist (IL-1Ra), a naturally occurring cytokine which acts as an inhibitor of IL-1β [11]. IL-1Ra binds to the IL-1 receptor (IL-1R) on ECs and prevents the binding of IL-1β to IL-1R [20]. Accordingly, various pro-inflammatory events, initiated by IL-1β:IL-1R binding, including the synthesis and releases of chemokines and enhanced influx of leukocytes in inflamed cornea, will be inhibited by Regener-Eye containing IL-1Ra [11]. Importantly, the IL-1Ra-dependent inhibition of the IL-1β:IL-1R axis results in the reduced generation of inflammatory CD4 + Th17 cells, which crucially contributes to the alleviation of Th17-cell-driven chronic keratitis [16,21]. d-MAPPS™ significantly attenuated neurotrophic keratitis in an 80-year-old patient [13]. Complete corneal healing was observed after only 15 days of d-MAPPS™-based treatment [Figure 2]. The therapeutic potential of d-MAPPS™ in the treatment of neurotrophic keratitis and corneal wounds relied on its capacity to: (i) improve the viability of injured HCECs, (ii) restore the morphology and function of corneal epithelium, and (iii) prevent the development of corneal inflammation [16,21]. Patients with corneal injuries should use d-MAPPS™ in order to enhance the repair and regeneration of the corneal epithelial barrier and to prevent the development and progression of keratitis.

### 2.2. Therapeutic Potential of d-MAPPS™ in the Treatment of Uveitis

Uveitis (inflammation of the iris, ciliary body, and choroid) is developed as a consequence of the detrimental immune response in the eye [4]. Microbial pathogens which by-pass the epithelial barrier activate resident DCs and macrophages [22]. Activated DCs and macrophages produce inflammatory cytokines which generate strong and efficacious protective Th1/Th17-cell-dependent adaptive immune responses in the inflamed uvea [4,22,23]. DC- and macrophage-derived IL-1β, TNF-α, and IL-12 have the most important pathogenic roles in the development of uveitis [4,22,23]. DC-derived IL-12 activates T-bet and STAT-4 transcriptional factors in naïve T cells, resulting in the generation of Th1 sub-populations of CD4+ and CD8+ T lymphocytes [4]. DC-sourced IL-1β, IL-6, IL-23, and TGF-β induce the generation and expansion of Th17 lymphocytes through the activation of RORyT and STAT-3 transcriptional factors in naïve T cells [4]. Macrophage-derived IL-1β acts synergistically with DC and macrophage-sourced TNF-α to induce the enhanced expression of E and P selectins on EC, enabling the massive recruitment of monocytes, CD4 + Th1, and Th17 lymphocytes in inflamed uvea [22]. CD4 + Th1 cells, in an IFN-γ-dependent manner, induce the activation of inflammatory M1 macrophages which, through the release of NO and ROS, aggravate acute uveitis [22,23]. CD4 + Th17 cells produce IL-17 and IL-22, which activate N1 neutrophils and innate lymphoid cells, playing important roles in the development and progression of chronic uveitis [24].

d-MAPPS™ contains soluble receptors of tumor necrosis factor alpha (sTNFRI, sTNFRII) and growth-related oncogene gamma (GRO-γ), which are able to prevent IL-1β-, TNF-α-, and IL-12-driven uveitis [11,16]. d-MAPPS™ containing sTNFRI and sTNFRII binds to the TNF-α and prevent the TNF-α-dependent over-expression of E and P selectins on EC, crucially contributing to the reduced recruitment of circulating monocytes and lymphocytes in the uvea of d-MAPPS™-treated eyes [11,16]. Significantly attenuated concentrations of IL-12 and IFN-γ were observed in the supernatants of d-MAPPS™-treated monocytes and lymphocytes [12]. d-MAPPS™ containing GRO-γ is able to suppress DCs:T cell cross-talk and efficiently inhibits the DC-dependent generation of inflammatory Th1 cells [12]. GRO-γ-treated DCs had a tolerogenic phenotype characterized by the increased secretion of immunosuppressive IL-10, and reduced the production of inflammatory cytokines IL-12, IL-23, IFN-γ, and TNF-α [12]. Accordingly, it is strongly expected that the intraocular administration of d-MAPPS™ would affect the cross-talk between IL-12- and IL-23-producing DCs and naive CD4+ T cells in lymph nodes, preventing the DC-dependent generation of IFN-γ-producing Th1 and IL-17-producing Th17 lymphocytes. The reduced presence of inflammatory Th1 and Th17 cells is crucial for the inhibition of M1 macrophages and N1 neutrophils, which will result in the diminished production of M1 macrophages and N1 neutrophil-sourced NO, ROS, and inflammatory cytokines, and will lead to the alleviation of uveitis. In line with these findings, d-MAPPS™ could be considered as a potentially new therapeutic agent in the treatment of uveitis.

### 2.3. Therapeutic Potential of d-MAPPS™ in the Treatment of Meibomian Gland Dysfunction and DED

Through the production of lipid-rich meibum that reduce aqueous tear evaporation, dysfunction meibomian glands provide tear film stability and protect the ocular surface against desiccation [25]. Accordingly, both congenital and acquired meibomian gland (MGD) result in increased tear film osmolarity and lead to the development of evaporative DED [26].

Dryness, grittiness, scratchiness, soreness, irritation, and burning are the most common symptoms reported by patients suffering from MGD [25,26]. Currently, there is no cure for MGD, and the treatments are directed towards improving the symptoms rather than towards eliminating the cause of the disease [27].

Hyperkeratinization of the ductal epithelium, obstruction of the meibomian gland orifice, and the inflammation-driven exhaustion of meibomian stem cells are considered as the most important pathogenic mechanisms responsible for the development of MGD [28]. d-MAPPS™ containing sTNFRI, sTNFRII, and IL-1Ra may prevent inflammation-dependent meibomian gland dropout [13]. Accordingly, d-MAPPS™ significantly attenuated MGD-related symptoms and remarkably improved the quality of life of a patient suffering from MGD [13,16]. d-MAPPS™ restored the morphology and structure of meibomian glands in a 55-year-old MGD patient. Meibomian ducts were dilated and meibomian glands were enlarged and tortuous with abnormal structures before the topical administration of d-MAPPS™. After three weeks of d-MAPPS™-based therapy, meibomian glands of the same patient exhibited a normal morphology and structure. The presence of hypoilluminescent grape-like clusters of meibomian glands, hyperilluminescent ducts, and tarsus indicated the beneficial effects of d-MAPPS™ in the treatment of MGD. Additionally, significantly improved tear film breakup time (TBUT), which was noticed three weeks after d-MAPPS™-based treatment, confirmed the restoration of meibomian gland function in d-MAPPS™-treated MGD patients [13,16]. Complications such as ocular pain, persistent bleeding, and infections were not observed during or after the administration of d-MAPPS™. Additionally, MGD patients did not report any adverse effects related to the administration of d-MAPPS™, indicating that d-MAPPS™ is safe for topical application in MGD patients [13,16].

MGD and chronic inflammation in the eye usually result in the development of DED [26]. Activated macrophages and neutrophils produce NO, ROS, and tumor necrosis factor alpha (TNF-α), which induce corneal nerve damage, leading to the development of DED in the medium-to-long term. Additionally, Th1- and Th17-cell-driven immune responses have important roles in the pathogenesis of DED [4,29]. Th1-cell-derived IFN-γ promotes apoptosis and squamous metaplasia of the ocular surface epithelia, whereas Th17-cell-sourced IL-17 disrupts epithelial cell barriers [4,29].

The MGD-dependent loss of homeostasis, instability, and hyperosmolarity of the tears are usually manifested by visual disturbance, dryness, grittiness, scratchiness, soreness, irritation, burning, watering, and eye fatigue [25,26]. Significantly reduced functional visual acuity impairs the performance of vision-dependent daily activities (such as reading, writing, and driving), greatly diminishing the quality of life of DED patients [30]. Currently, there is no cure for dry eyes, and the treatments are directed towards improving the symptoms in order to break the vicious circle of chronic inflammation [30,31]. Therefore, the main treatment strategy has shifted from hydration and lubrication of the dry ocular surface to local, intraocular immunosuppression [31,32,33].

We recently demonstrated the beneficial effects of d-MAPPS™ in DED treatment [13]. d-MAPPS™ significantly efficiently alleviated ocular symptoms (pain, dryness, grittiness, scratchiness, soreness, irritation, burning, watering, and eye fatigue) in 131 DED patients [13]. Visual analogue pain scores (VAS) and Standard Patient Evaluation of Eye Dryness Questionnaire (SPEED) scores were significantly attenuated in d-MAPPS™-treated DED patients during the one year of follow-up [13]. Immunomodulatory properties of d-MAPPS™ were mainly responsible for its beneficial effects in DED treatment [11,12,13,16]. d-MAPPS™ modulated the phenotype and function of all immune cells which play important pathogenic roles in the progression of DED [12]. d-MAPPS™, in GRO-γ- and IL-1Ra-dependent manners, suppressed the production of pro-Th1 and pro-Th17 cytokines (IL-12, IL-1β, and TNF-α) in activated DCs and prevented the DC-dependent generation of Th1 and Th17 cells [12]. Additionally, d-MAPPS™, in sTNFRI-, sTNFRII-, and IL-1Ra-dependent manners, inhibited the production of IFN-γ in Th1 cells, IL-17 in Th17 cells, and TNF-α and IL-1β in macrophages [12]. By suppressing the production of these inflammatory cytokines, which are generally considered responsible for corneal nerve damage and disruption of the ocular epithelial barrier in DED patients, d-MAPPS™ prevented the aggravation of ongoing inflammation and enabled the creation of an immunosuppressive microenvironment which enhanced the repair of injured tissue [13]. CD4 + CD25 + FoxP3 + T regulatory cells (Tregs) have a crucially important immunosuppressive role in DED [34]. A reduced number of Tregs is observed in DED patients with aggravated diseases, while increased numbers of Tregs are followed by tissue repair and regeneration [35,36]. Indoleamine 2, 3-dioxygenase 1 (IDO1) maintains populations of Tregs in inflamed tissue by preventing their transdifferentiation in inflammatory Th1 and Th17 cells [37,38]. IDO1 activates general control nonderepressible 2 (GCN2) kinase in activated Tregs, which inhibits mTOR signaling and prevents the destabilization of immunosuppressive phenotypes of Tregs, enabling their expansion [39]. Considering that elevated IDO1 activity was measured in d-MAPPS™ [11], we believe that the IDO-1-dependent expansion of Tregs could be, at least partially, responsible for the beneficial effects of d-MAPPS™ in DED patients.

There is a causal relationship between wearing contact lenses and developing DES. Contact lens wear induces meibomian gland dropout and obstruction of the meibomian gland orifice [40,41]. Mechanical friction, caused by contact lenses, induces injury and morphological changes in conjunctival epithelial cells. Additionally, contact lens wear induces meibomian gland dropout and obstruction of the meibomian gland orifice [40,41]. The majority of the 140 million contact lens wearers worldwide report dry-eye-related symptoms which are usually manifested late in the day, when they impair the performance of vision-dependent activities [41]. We recently demonstrated that d-MAPPS™ managed to efficiently alleviate dry-eye-related symptoms in contact lens wearers. Importantly, the topical administration of d-MAPPS™ minimized contact lens discomfort without causing any side effects [13,16]. Due to its capacity to promote corneal repair and regeneration and due to its potent immunoregulatory properties, d-MAPPS™ should be used as a new therapeutic agent for the treatment of DES in contact lens wearers.

### 2.4. Therapeutic Potential of d-MAPPS™ in the Treatment of Th1- and Th17-Cell-Driven Autoimmune Diseases with Ocular Manifestations

d-MAPPS™ contains many anti-inflammatory and immunosuppressive factors (IL-1Ra, sTNFRI, sTNFRII, and GRO-γ) which are able to efficiently suppressed detrimental Th1- and Th17-cell-driven immune response in the eyes of DED patients [11,12,13]; d-MAPPS attenuates inflammation in the eye of DED patients through the immunoregulatory effects of IL-1Ra and sTNFRs, which inhibit IL-1β- and TNF-α-driven inflammation and through the GRO-γ-dependent suppression of eye-infiltrated inflammatory Th1 and Th17 lymphocytes [11,12]. d-MAPPS containing IL-1Ra is an anti-inflammatory cytokine which binds to IL-1R on ECs [11]. In this way, IL-1Ra prevents the enhanced expression of E and P selectins elicited by the binding of inflammatory IL-1β to the EC’s IL-1R [11]. The IL-1ra-dependent downregulation of E and P selectins reduces the influx of activated neutrophils, monocytes, and lymphocytes in injured tissues [12]. Similarly, d-MAPPS containing sTNFRs bind to TNF-α and inhibit its biological and inflammatory effects, particularly the TNF-α-driven recruitment of leukocytes in inflamed eyes [11,12]. GRO-γ induces the generation of immunosuppressive and tolerogenic phenotypes in monocyte-derived DCs. Tolerogenic DCs have an impaired capacity for antigen presentation and the activation of naive T cells [11]. Accordingly, through the immunosuppressive effects of GRO-γ, d-MAPPS attenuates the antigen-presenting properties of DCs and prevents the DC-dependent generation of inflammatory Th1 and Th17 cells [11,12]. Therefore, d-MAPPS™ should be considered a new therapeutic agent for the treatment of Th1- and Th17-cell-driven autoimmune diseases with ocular manifestations, including systemic lupus erythematosus (SLE), rheumatoid arthritis (RA), and Sjogren’s syndrome (SS). An increased presence of inflammatory, IFN-γ-producing Th1 and IL-17-producing Th17 cells, accompanied with a reduced number of immunosuppressive Tregs, were observed in the eyes of patients suffering from these autoimmune diseases [4,9]. The long-term use of immunosuppressive drugs and corticosteroids is limited due to the serious side effects and possible development of glaucoma and cataracts [10]; therefore, d-MAPPS™ could be used for the safe and efficient attenuation of autoimmune responses in the eyes of SLE, RA, and SS patients. Indeed, the reduced expansion of inflammatory Th1 and Th17 cells and an increased generation of immunosuppressive Tregs were observed in a population of peripheral-blood-derived mononuclear cells activated in vitro which were cultured in the presence of d-MAPPS™ [12], confirming its therapeutic potential in the treatment of Th1- and Th17-cell-driven autoimmune diseases with ocular manifestations.

Platelet factor 4 (PF4), which is present in high concentrations in d-MAPPS™, limits the generation of Th17 cells and is crucially involved in the suppression of Th17-cell-driven inflammation in the eyes of SLE, RA, or SS patients [11,42]. Additionally, d-MAPPS™ may activate tissue-resident mesenchymal stem cells (MSCs) which, due to their immunosuppressive properties, may enhance the anti-inflammatory immune response in patients with autoimmune diseases [15]. d-MAPPS™ contains high concentrations of IL-27, which induces the generation of immunoregulatory phenotypes in MSCs in injured and inflamed eyes [11]. d-MAPPS containing IL-27 promotes the immunosuppressive potential of MSCs by enhancing the MSC-dependent generation of IL-10-producing CD4 + T cells within the population of activated helper T cells [43]. Additionally, the capacity of MSCs to induce the apoptosis of inflammatory Th1 and Th17 cells in a programmed death ligand 1 (PDL1)-dependent manner is significantly enhanced by IL-27 [43].

### 2.5. Therapeutic Potential of d-MAPPS™ in the Treatment of Idiopathic Orbital Inflammatory Syndrome

Idiopathic orbital inflammatory syndrome (IOIS) represents a heterogeneous group of disorders characterized by orbital inflammation without any identifiable local or systemic causes [44]. In adults, IOIS is usually unilateral, but children more commonly present with bilateral disease. Clinical presentation involves pain in the eye, redness, chemosis, proptosis, and periorbital edema [44]. Although corticosteroids are very effective in the therapy of acute IOIS, approximately 37% of steroid-treated patients showed failure to resolve ongoing inflammation [10]. Patients with steroid-non-responsive IOIS require treatment with multiple systemic immunosuppressant drugs (such as methotrexate and cyclophosphamide) and radiotherapy [10,44]. Infliximab, a monoclonal antibody which binds to TNF-α, is used as a new therapeutic agent in the treatment of steroid-non-responsive IOIS [45]. Clinical improvement, noticed in Infliximab-treated patients, suggests that TNF-α is crucial for the progression of IOIS [44,45]. However, the long-term use of Infliximab increases the risk for the reactivation of tuberculosis and the possible development of lymphoma and indicates the use of new biologics which should suppress TNF-α-driven inflammation in IOIS patients without causing life-threatening systemic immunosuppression [45].

d-MAPPS™ contains sTNFRI and sTNFRII, which bind to TNF-α and prevent interactions between TNF-α and membranous TNFRI, which is expressed on the surface of ECs in the inflamed eyes of IOIS patients [11]. Molecular mechanisms responsible for the beneficial effects of sTNFRI involve the suppression of two main signaling pathways elicited by TNF-α–TNFRI interactions: the NF-κB and Janus kinase (JNK) pathways [11,16]. NF-κB is a transcription factor that mediates the transcription of proteins which regulate proliferation and the inflammatory response in immune and parenchymal cells [11,16]. TNF-α activates the JNK-inducing kinases which phosphorylate signal regulatory protein kinase 2 (SEK2) which, in turn, activates JNK [16]. JNK translocates to the nucleus and activates transcription factors which promote the synthesis of inflammatory cytokines and chemokines [16]. Accordingly, the therapeutic potential of d-MAPPS™ in the treatment of IOIS relies on the sTNFRI- and sTNFRII-based inhibition of NF-κB- and JNK-driven inflammation in the eyes of IOIS patients [11].

### 2.6. Therapeutic Potential of d-MAPPS™ in the Treatment of Herpes-Virus-Induced Eye Inflammation

Herpes viruses may cause direct or indirect injury (due to the excessive and aberrant activation of immune response) of the epithelia and ECs in the eyes [46,47]. Vision loss, which occurs when hemorrhages, scars, edema, or vascular occlusion involve the fovea and/or macula, could be prevented by the timely and potent activation of NK cells and herpes-virus-specific T lymphocytes which efficiently eliminate viral infected cells [47]. d-MAPPS™ contains several lymphocyte and monocyte-attracting chemokines (CXCL16, CCL21, and CXCL14) which attract CD8+ cytotoxic T lymphocytes (CTLs) and NK cells, which are the immune cells that play crucially important roles in the elimination of viral pathogens [11]. d-MAPPS™ containing CXCL16 binds to the CXCR6 receptor which is highly expressed on memory CTLs and cytotoxic NK cells [11]. Additionally, d-MAPPS™ contains high concentrations of CCL21, which serve as the ligand for CCR7, expressed on naïve T cells and activated DCs [11]. The CCL21:CCR7 axis is the most important signaling cascade which regulates the recruitment of naïve T cells and activated DCs in peripheral lymph nodes, enabling their cross-talk [4]. Accordingly, CCL21-containing d-MAPPS™ will enhance the activation of herpes-virus-specific CD8 + T cells in the periorbital region and promote the generation of anti-viral, T-cell-driven immune responses in the eyes of herpes-virus-infected patients [16]. In line with these findings, it is highly expected that d-MAPPS™-based therapy will improve the intraocular immune response against herpes viruses, resulting in the attenuation of herpes-virus-induced eye inflammation.

### 2.7. Therapeutic Potential of d-MAPPS™ in the Treatment of Retinal Diseases

Retinal detachment and consequent photoreceptor cell results in the vision impairment and are considered as the main causes of retinal dysfunction [48]. Both autophagy and retinal inflammation, particularly elevated levels of TNF-α, participate in photoreceptor cell death after retinal detachment [48]. TNF-α induces the increased expression of pro-apoptotic and autophagy-related genes in photoreceptor cells, and crucially contributes to their increased loss. The use of a TNF-α inhibitor (Infliximab) modulated the autophagy and apoptosis-related signaling pathways, which resulted in the enhanced survival of photoreceptor cells [48]. An inhibition of the TNF-α-dependent apoptosis of photoreceptor cells is crucially responsible for the repair and regeneration of injured retinal tissue [48]. d-MAPPS™ contains high concentrations of sTNFRI and sTNFRII, which suppress the TNF-α-driven injury of photoreceptor cells [11,48]; therefore, the topical administration of d-MAPPS™ could be considered as a new therapeutic approach which would increase the survival of photoreceptor cells and contribute to the repair of injured retinal tissue.

Although retinitis pigmentosa (RP) is considered a genetic disorder of the retinal tissue, chronic inflammation also plays an important pathogenic role in the progression and aggravation of this disease [49]. Strong inflammatory reactions and massive influxes of inflammatory cells were observed in the anterior vitreous cavities of RP patients [49]. Significantly increased concentrations of inflammatory chemokines (monocyte chemotactic protein-1) and cytokines (pro-Th1 (IL-12 and TNF-α) and pro-Th17 (IL-1β, IL-6, and IL-23) were measured in the aqueous humor and vitreous fluid of RP patients [49,50]. d-MAPPS™ containing GRO-γ, IL-1Ra, sTNFRI, and sTNFRII suppress IL-12-, IL-1β-, and TNF-α-dependent inflammation and inhibit Th1- and Th17-cell-driven immune responses in the eye [11,12,13]. Accordingly, it is strongly expected that the topical administration of d-MAPPS™ would prevent the inflammation-dependent aggravation of RP and could be considered as an adjuvant therapeutic agent in RP treatment.

It is well known that hyperglycemia impairs the retinal microvasculature, which leads to a continuous decrease in retinal blood flow and results in the ischemic injury of retinal cells [51]. Additionally, inflammatory cytokines (TNF-α, IL-1β, and IL-6) produced by CD68-expressing macrophages recruit inflammatory immune cells in the retinas of diabetic animals, crucially contributing to the development and progression of diabetic retinopathy [51]. The massive injury of retinal cells, severe edema, extensive hemorrhage, and abnormal thickness of all retina layers (ganglionic layer (GL), inner plexiform layer (IPL), outer plexiform layer (OPL), and outer nuclear layer (ONL)), observed in diabetic animals were accompanied with elevated levels of TNF-α and IL-1β in their vitreous bodies [51]. d-MAPPS™ contains high concentrations of sTNFRI, sTNFRII, and IL-1Ra [11]. Accordingly, the topical administration of d-MAPPS™ could result in the inhibition of TNF-α- and IL-1β-driven inflammation in retinal tissue and may be crucial in the prevention of diabetic retinopathy. Therefore, d-MAPPS™ should be considered as a new therapeutic agent in the treatment of diabetic retinopathy.

## 3. Conclusions

d-MAPPS™ is enriched with immunomodulatory and trophic factors which efficiently suppress detrimental immune responses in the eye and promote the repair and regeneration of injured corneal and retinal tissues [11,12,13,14,15,16]. d-MAPPS™ increased the viability of injured corneal cells, inhibited the production of inflammatory cytokines in immune cells, alleviated inflammation, and restored the vision loss of patients suffering from MGD and DED [11,12,13]. Therefore, the topical administration of d-MAPPS™ could be considered as a new therapeutic approach for the treatment of ocular inflammatory diseases and for the repair and regeneration of injured corneal and retinal tissues.

## Data Availability

Not applicable.
